# Parental Stress and Quality of Life in Parents of Young Children with Autism

**DOI:** 10.1007/s10578-024-01693-3

**Published:** 2024-05-09

**Authors:** Leanne Dijkstra-de Neijs, Daphne B. Boeke, Ina A. van Berckelaer-Onnes, Hanna Swaab, Wietske A. Ester

**Affiliations:** 1 Sarr Autism Rotterdam, Youz Child- and Adolescent Psychiatry, Parnassia Group, Dynamostraat 18, 3083 AK Rotterdam, The Netherlands; 2 Clinical Neurodevelopmental Sciences, Leiden University, Leiden, The Netherlands; 3 Parnassia Group, Parnassia Academy, Den Haag, The Netherlands; 4 Curium-LUMC, Department of Child- and Adolescent Psychiatry, Leiden University, Oegstgeest, The Netherlands; 5 Leiden Institute for Brain and Cognition, Leiden University, Leiden, The Netherlands

**Keywords:** Parental stress, Quality of life, Autism, Young children

## Abstract

Parents of children with ASD are at risk for chronic stress due to challenging parenting. It is unknown whether stress is already present in early parenthood, similar for mothers and fathers and if this impacts quality of life (QoL). Parental stress and QoL were assessed in 56 mothers and 51 fathers of young children (aged 3 to 7) with autism. Associations between parental stress (OBVL) and QoL (WHOQoL-BREF) were examined. Parents of young children with ASD appear to have high parental stress from conflicting feelings towards their child and from difficulties with parenting. Mothers have higher stress from feeling confined in their motherly role than fathers compared to the OBVL norm population. Both mothers and fathers have a low QoL. Increased maternal conflicting feelings towards the child associated with lower psychological QoL, while high maternal feelings of role confinement associated with low physical QoL. Increased paternal conflicting feelings towards their child related to lower physical and social QoL, while fathers with more parenting difficulties reported less satisfaction with their psychological and environmental wellbeing. Thus, already at young age, parenting children with ASD is a major challenge for both mothers and fathers.

## Introduction

Parents of children with ASD are at risk for chronic stress due to challenging parenting [14, 27], which may affect their quality of life (QoL). Most of the parenting stress-related research has been conducted in mothers of school aged children and less is known about the stress of parents of young children with ASD, the stress levels of fathers, and the impact of their parenting stress on QoL.

It is known that mothers of school aged children with ASD experience 1.5-times higher levels of daily parenting stress [27], present more stress-related mental and/or physical problems and have an elevated mortality hazard ratio compared to mothers of typically developing children [20, 27, 40, 41, 46, 58]. Increased parental stress in children with ASD is found to be related to attuning to the child’s special needs [32, 40]. In addition, the lack of adequate reciprocity, a primary symptom of autism, may result in parents experiencing the interaction with their child as emotionally challenging, compared to parents with typically developing children [2, 34, 40, 55]. Although parental stress has been extensively studied as an independent, outcome and mediator variable in ASD research and recent studies have shown that parents of children who have not yet been diagnosed with ASD already experience increased stress levels compared to parents of typically developing children [12, 52], the question is still underexposed whether parental stress is already present in parents of young children with ASD. During this stage of life, the external demands are less challenging for the child than at a later age when developmental challenges become more complex. The few studies that did address parental stress at an early age, showed that parents, mostly mothers, of children with ASD aged 2 to 5 report more overall parenting stress than parents of typically developing children or children with a developmental delay. Therefore, more attention is needed to explore the mechanisms inducing stress [17–19, 23]. Parenting demands may not only lead to high parental stress, it can also lead to more pronounced behavioral problems in children [22, 45, 57], due to children’s response to parental stress. Conversely, behavioral problems in the child can induce parental stress [39, 57]. It is therefore crucial to obtain better insight into parental stress in parents with young children with ASD. Associated to the question about parental stress in parents of young children with ASD, is the question of the impact of raising a young child with ASD on the QoL in their parents. Studies in parents of older school aged children show that parental stress is associated with a decreased overall QoL [13, 30, 53]. Specifically, family functioning [31] and parental physical wellbeing are affected [29].

Most studies on parental stress in children with ASD focus on mothers, and not on fathers [21]. Consequently, much less is known about fathers’ coping strategies while parenting a child with ASD [43]. Results of studies of parents raising children with ASD that do include fathers are often based on small sample sizes, do not differentiate for gender during analyses or include parents from different families [24], hindering comparison between fathers and mothers. The few studies that did explore the differences between mothers and fathers, found varying results [3, 21, 24, 38, 42, 47]. Learning about differences between parental experiences of fathers and mothers may help to understand mechanisms regarding parent factors that impact parental mental well-being and consequently also the well-being of their children.

With respect to parental stress, it is important to understand the impact of specific stress inducing factors, like interaction related factors or factors related to the impact of the extra effort that is needed from parents, and their effect on different domains of parental QoL in both mothers and fathers of young children with ASD. The current study aims to examine the question whether high parental stress and low QoL is already present in both mothers and fathers of young children with ASD and explores whether maternal and paternal stress impact parental QoL.

## Methods

### Study Design

The present study is a descriptive, correlational, cross-sectional study assessing parental stress and QoL in parents of young children with ASD. This study is part of the ongoing Tandem study (Dutch trial register: NL7534) [36], approved by the Institutional Review Board of Leiden University Medical Center, The Netherlands (NL6378.058.18).

### Participants

Parents were eligible for inclusion if (1) their child was diagnosed with ASD, and (2) the child was aged 3 to 7 years. Parents were recruited from Youz Parnassia Group and GGZ Delfland, both mental health care providers in the Netherlands. Parents eligible for inclusion were asked by their child's psychologist for consent to be contacted by the research team shortly after their child being diagnosed with ASD. If parents agreed, contact details of the parents were passed on to the research team. The research team then screened the parents by telephone to verify whether they met the inclusion criteria and if they were willing to participate. If they did, an informed consent meeting took place where parents received detailed information about their study participation and signed an informed consent form. After informed consent, a research employee provided parents with a link with which they could enter explanation about the study and complete their questionnaires within Research Manager, a data management program. If parents had questions about the questionnaires, they could contact an employee of the research team by mail or telephone. No incentives for participation were given. In total 107 parents (56 mothers and 51 fathers) of 61 children were recruited.

### Measures

#### Parental Stress

Parental stress was assessed by self-report on three areas of parental stress using the Dutch parenting burden questionnaire (in Dutch: *Opvoedbelasting Vragenlijst*, OBVL [51]). This questionnaire assesses stress related to (1) parental conflicting feelings towards the child*,* represented by the degree to which the parent has compromised feelings/thoughts about the child for example ‘I feel happy with my child’ (6 items); (2) difficulties with parenting, concerning the extent to which the parent feels they doubt to have enough skills in the upbringing of their child, for example ‘My child listens to me’ (7 items), and (3) the extent to which the parent experiences the parental role as confining, for example ‘Because of my child's upbringing, I don't get enough time for myself’ (6 items) [51]. The OBVL uses a 1–4 response scale (1 = does not apply, 2 = does somewhat apply, 3 = does apply, 4 = totally does apply). Raw scores are transformed into T-scores which give clinical meaning using norm tables, as presented by Vermulst et al. [51] (see “Appendix 1”). Explorative factor analysis has shown that the three theoretically assumed parental stress areas within the OBVL are empirically demonstrated [51]. The concept validity of the three parental areas is good. The norm population used within this study is the OBVL norm population [51]. The norm population included data on 848 mothers of typically developing Dutch children (50.8% boys) from the general population [51].

#### Quality of Life

QoL was assessed using parental self-report on four scales of the World Health Organization Quality of Life (abridged version) questionnaire (WHOQoL-BREF), including: (1) ‘physical health’, incorporating activities of daily living, energy and mobility (7 items); (2) ‘psychological health’, incorporating bodily image, feelings and self-esteem (6 items); (3) ‘social relationships’, including personal relations, social support and sexual activity (3 items); (4) ‘environment’, including financial resources, health- and social care and home environment (8 items) [56]. The WHOQoL-BREF is frequently used to assess QoL in a variety of situations and populations [48]. This self-report questionnaire includes 26 items with a 1–5 response scale (1 = not at all, 2 = almost not, 3 = average, 4 = pretty good, and 5 = completely). An item example is: “How much do you enjoy life?” A higher domain score (min = 1 and max = 100) corresponds to a better QoL. Clinical outcomes on the WHOQoL-BREF can be interpreted as “excellent”, “very good”, “good”, “fair” or “poor” satisfaction with someone’s QoL, see “Appendix 2” [26]. Internal consistency, item–total correlations, discriminant validity, and construct validity through confirmatory factor analysis indicate good to excellent psychometric properties of reliability and tests of validity [44, 48]. The WHOQoL-BREF is a cross-culturally valid assessment but unstandardized questionnaire of QoL [44, 48]. Normative values obtained in the general population of the same culture preferably serve as norm population (Fayers & Machin, 2007). Since only an Australian norm population is available and QoL ratings within the Dutch and Australian population are similar this comparison does provide insights into the QoL of life of the parents within our study population. The norm population scores are based on the general Australian population recruited through random telephone selection and weighted by socio-economic status (n = 396, 64% males, mean age of 48.2 years, s.d. = 17.3). Norm values are present for women and men [26]. The questionnaires were scored using a manual-based syntax in SPSS.

### Covariates

*Social demographic markers* (marital status, education, financial situation, country of origin) are measured with a self-reported social-demographic questionnaire. The questions were: (1) What is your marital status (single, cohabiting/married, (2) What is your level of completed education (primary education/lower pre-vocational education, secondary education/secondary vocational education, higher vocational education/academic education), (3) What is your income (below average/less than 2800 euros per month, average/around 2800 euros/month, above average/more than 2800 euros/month), (4) What is your country of birth.

*Psychological distress* is measured with the Brief Symptom Inventory (BSI; [10]). The BSI consists of 53 items and is the abbreviated version of the Symptom Checklist 90 (SCL-90). Within this study, the BSI total scale was used, which measures the severity of psychological distress. The reliability of the total BSI scale is good (α = 0.96, inter-item correlation = 0.32, test–retest correlation = 0.90), the validity is supported by good discriminatory power between patients and healthy respondents as well as between patients’ groups with different diagnoses. Questions that are addressed within the BSI include: How much trouble did you have in the past week from: ‘being quickly annoyed by something’ or ‘being anxious in open squares or large spaces’ [5, 10]).

### Statistical Analysis

Social demographic markers (marital status, education, financial situation, country of origin) and psychological distress (BSI) were compared to the general population using Pearson χ^2^ test, to determine which variable should be included within analysis as covariates.

To evaluate parental stress weighted means and pooled standard deviations for the three parental stress areas (parental conflicting feelings, difficulties with parenting and role confinement) in the study population were calculated. Mothers raw scores from the study population were compared to mothers of the norm population using Welch’s *t*-tests [11, 51]. The differences between mothers from the study population and the norm population on the three areas of the OBVL are determined by comparing the T-scores of the study population to the clinical cut-off T-scores scores provided by the OBVL manual, see “Appendix 1” [51]. Since no norm scores for fathers are available, raw scores on the OBVL of mothers from the study population are compared to raw scores of fathers from the study population using Welch’s *t*-tests to explore whether there is a difference between mothers and fathers. To report on parental stress scores, *z*-scores were calculated based on mothers of typically developing children [1].

To explore the QoL outcomes in the four QoL domains of the WHOQoL-BREF (physical, psychological, social and environment) mothers and fathers within the study population are compared with women and men of the norm population [26] using Welch’s *t*-tests. The differences between the study population and the norm population are determined by comparing domain scores of the mothers and fathers of the study population with the women and men of the norm population [26]. Scores are considered clinically relevant when they are within the ‘poor’ category of the norm population (“Appendix 2”).

To study the relation between parental stress areas and QoL domains linear regressions for both mothers and fathers were performed on raw scores of all variables. During step one the enter method was used wherein the covariates in which the study population significantly differed from the norm population were entered. In step two forward selection was used wherein the parental stress area that had the highest correlation with the parental QoL domain was entered first. By this model, we examined which of the parental stress areas were a significant additional predictor of parents’ QoL when the covariates were corrected, regardless of whether they were significantly different. Correlations were performed between the raw scores of the OBVL and the WHOQoL-BREF raw scores. For all analyses, the statistical criterion for entry was a probability of *p* < 0.05. When no effect between the independent (parental stress) and dependent (QoL) variables was present, it was examined whether (1) there was an interaction effect, as one of the covariates could influence this relationship or (2) there was curvilinearity, as this type of relationship wherein the pattern of association between the dependent and independent variables change as the values of the variables change is not detected by linear regression analysis. Analyses were performed using Statistical Package for the Social Sciences (SPSS) version 25.0.

## Results

### Descriptive Statistics

A total of 184 families [children with their parent(s)] were eligible to participate in this study. Of these 184 families, 130 families allowed to be approached by the research team after being asked by their therapists. 70 families of these130 indicated that they wanted to participate and signed informed consent. However, 9 of the 70 families decided before the start of the study that they could not participate because of lack of time. The included 61 families comprised out of 107 parents (mothers: n = 56, mean age = 34.44 years, *s.d.* = 4.69; fathers: n = 51, median age = 37.0 years, *IQR* = 6.0) of 61 children with ASD (mean age = 4.6 years, *s.d.* = 1.1, 55 boys). Within the current study population three times as few single fathers were present than in the norm population [χ^2^(1) = 7.33, *p* < 0.01]*.* Both mothers and fathers were higher educated than the Dutch general population [mothers: *χ*^2^(2) = 14.0, *p* < 0.001, fathers*: χ*^2^(2) = 7.23, *p* < 0.03]*.* More than half of the fathers in the study population had an above-average salary, which is higher than fathers of the Dutch population [*χ*^2^(2) = 10.36, *p* < 0.01]*.* Both mothers and fathers reported more psychological distress [mothers: χ^2^(2) = 60.6*, p* < 0.01, fathers: χ^2^(2) = 20.6*, p* < 0.01] than the norm population (Table 1).

**Table 1:** Descriptives of the participating parents and their young children with ASD within this study compared to the Dutch population

	Parents of child with ASD		ASD parents vs. comparison group	χ^2^	*p*	Comparison group
	N	%	Expected %			
Marital status						
Mothers						
Single	10	19.2	23.0	0.42	NS	Dutch families (n = 798,200) with children living at home < 18 year in 2022 [16] (https://www.nji.nl)
Living together/married	42	80.8	77.0			
Fathers						
Single	3	6.3	23.0	7.33	< 0.01	Dutch families (n = 798,200) with children living at home < 18 year in 2022 [16] (https://www.nji.nl)
Living together/married	44	93.6	77.0			
Education level						
Mothers						
Low	5	9.6	30.6	14.0	< 0.01	Dutch general population (n = 15,562,700) in 2020/2021 [8, 9] (https://opendata.cbs.nl)
Middle	23	44.2	41.7			
High	24	46.1	27.7			
Fathers						
Low	6	13.0	30.6	7.23	< 0.05	Dutch general population (n = 15,562,700) in 2020/2021 [8, 9] (https://opendata.cbs.nl)
Middle	22	47.8	41.7			
High	18	39.1	27.7			
Financial income						
Mothers						
Below average	24	51.1	47.4	0.41	NS	Dutch female population (n = 684,940) in 2020 [8, 9] (https://www.cbs.nl)
Average	14	29.8	34.2			
Above average	9	19.1	18.4			
Fathers						
Below average	8	17.8	28.8	10.36	< 0.01	Dutch male population (n = 633,830) in 2020 [8, 9] (https://www.cbs.nl)
Average	10	22.2	34.3			
Above average	27	60.0	36.7			
Psychological distress						
Mothers						
Normal	21	44.7	50.0	60.6	< 0.01	Standard population (n = 1206) from the BSI [5]
Sub-clinical	5	10.6	15.9			
Clinical	21	44.7	6.7			
Fathers						
Normal	29	65.9	50.0	20.6	< 0.01	Standard population (n = 1206) from the BSI [5]
Sub-clinical	2	4.5	15.9			
Clinical	13	29.5	6.7			
Country of origin						
Mothers						
Netherlands	42	79.2	73.8	0.51	NS	Total first- and second-generation immigrants (n = 2,241,220) in the Dutch population in 2022 [7] (https://www.cbs.nl)
Non-Dutch-Western	6	11.3	11.5			
Non-Dutch Non-Western	5	9.4	14.7			
Fathers						
Netherlands	41	87.2	74.0	2.93	NS	Total first- and second-generation immigrants (n = 2,241,220) in the Dutch population in 2022 [7] (https://www.cbs.nl)
Non-Dutch-Western	1	2.1	11.5			
Non-Dutch Non-Western	5	10.6	14.5			

*%* Percentage within the study or norm population, *NS* not significant

### Parental Stress

#### Parental Conflicting Feelings

Mothers of a young child with ASD experience more stress from conflicting feelings towards their child compared to mothers of typically developing children from the general population (*t* =  − 5.49, *p* < 0.01, *z* = 1.06, see Table 2). 51.8% Of the mothers’ report (sub)clinical stress from conflicting feelings towards their child. Mothers and fathers do not differ in experienced stress from their conflicting feelings towards their child (*t* = 0.41, *p* = 0.18).

**Table 2:** Parental stress in mothers of a young child with ASD compared to mothers of typically developing young children and fathers of a young child with ASD, measured with the parental stress questionnaire (OBVL)

Parental stress	Study population	Norm population	*p*	Study population	Study population	*p*
	Mothers	Mothers		Mothers	Fathers	
	(n = 56)	(n = 848)		(n = 56)	(n = 51)	
	Mean (sd.)	Mean (sd.)		Mean (sd.)	Mean (sd.)	
Conflicting feelings	9.86 (3.27)	7.78 (2.17)	< 0.01	9.86 (3.27)	9.58 (3.85)	NS
Parenting difficulties	14.39 (3.61)	11.51 (2.94)	< 0.01	14.39 (3.61)	15.34 (4.64)	NS
Role confinement	13.50 (4.68)	9.53 (2.76)	< 0.01	13.50 (4.68)	10.93 (3.81)	< 0.01

The norm population including mothers of typically developing children is derived from the parental stress questionnaire (OBVL) manual [51]. To correct for the age difference between the study population (3 till 7 years) and norm population (0 to 3 years and 4 to 11 years), weighted means and pooled standard deviations are created for the norm population. The parental stress score for ‘conflicting feelings’ and ‘role confinement’ is min. 6 and max. 24. The parental stress score for ‘parenting difficulties’ is min. 7 and max. 24

*NS* not significant

#### Parenting Difficulties

Mothers experience more stress deriving from difficulties with parenting than mothers of typically developing children (*t* =  − 7.93, *p* < 0.01, *z* = 1.09, see Table 2). 55.4% Of the mothers’ experience (sub)clinical problems deriving from difficulties with parenting. Mothers and fathers display comparable parental stress from difficulties in parenting of their young child with ASD (*t* =  − 1.26, *p* = 0.08).

#### Role Confinement

Mothers show more parental stress from feeling confined in their role as a parent compared to mothers of typically developing children (*t* =  − 8.55, *p* < 0.001, *z* = 1.04, see Table 2). 55.4% Of the mothers’ experience (sub)clinical problems with role confinement. Mothers experience more stress in feeling confined in their role as a parent compared to fathers of a child with ASD (*t* = 3.25, *p* =  < 0.01, Table 2).

### Quality of Life

#### Physical QoL

Both mothers (*t* = 5.08, *p* < 0.01) and fathers (*t* = 6.70, *p* < 0.01) of a young child with ASD experience lower physical QoL compared to the norm population (see Table 3). 20.0% Of the mothers and 6.1% of the fathers experience poor physical QoL.

**Table 3:** Quality of Life in parents of a young child with ASD compared to the norm population measured with the WHOQoL-BREF

Quality of life	Study population	Norm population	*p*^b^	Study population	Norm population	*p*^c^
	Mothers	Women		Fathers	Man	
	n = 55	n = 481		n = 49	n = 376	
	Mean (sd.)	Mean (sd.)		Mean (sd.)	Mean (sd.)	
Physical QoL	56.99 (10.86)	72.1 (14.2)	< 0.01	63.80 (8.95)	75.8 (12.7)	< 0.01
Psychological QoL	58.95 (8.73)	69.1 (10.5)	< 0.01	61.69 (8.75)	70.3 (11.9)	< 0.01
Social QoL	57.31 (10.07)	70.9 (17.1)	< 0.01	55.18 (11.95)	69.8 (16.4)	< 0.01
Environmental QoL	61.94 (9.74)	74.6 (12.5)	< 0.01	63.06 (8.61)	75.4 (11.7)	< 0.01

^a^The norm population included a general population derived from Hawthorne et al. [26], providing one decimal

^b^Significant difference between mothers of the study population and females of the general population

^c^Significant difference between fathers of the study population and males of the general population

#### Psychological QoL

Both mothers (*t* = 5.34, *p* < 0.01) and fathers (*t* = 5.10, *p* < 0.01) of a young child with ASD experience lower psychological QoL compared to the norm population (see Table 3). Of the mothers, 38.2% report a poor psychological QoL. Of the fathers, 20.4% experience their psychological QoL as poor.

#### Social QoL

Both mothers (*t* = 5.04, *p* < 0.01) and fathers (*t* = 4.63, *p* = 0.01) report lower social QoL compared to the norm population (see Table 3). Of the mothers, 63.6% report poor social QoL and so do 44.9% of the fathers.

#### Environmental QoL

Both mothers (*t* = 6.17, *p* < 0.01) and fathers (*t* = 7.56, *p* < 0.01) of a young child with ASD experience lower environmental QoL compared to the norm population (see Table 3). Of the mothers, 47.3% report poor environmental QoL and 65.3% of the fathers experience their environmental QoL as poor (Table 3).

### Associations of QoL by Specific Parental Stress Areas

#### Mothers Physical QoL

During step one of the regression analysis the enter method was used wherein the covariates mother’s education level and mothers’ psychological distress were added. In step two forward selection was used wherein stress derived from feeling confined in their motherly role was entered first (*r* =  − 0.44, *p* < 0.01), followed by mothers’ conflicting feelings towards her child (*r* =  − 0.29, *p* < 0.05). Results show that 31% of the variance in mothers physical QoL could be explained mothers’ psychological distress [F(2,43) = 9.86, *p* =  < 0.01]. Another 7% of variance in mothers’ physical QoL could be explained by mothers’ stress derived from feeling confined in their motherly role [F(1,42) = 5.02, *p* = 0.03]. The best fitting models were found for mothers’ role confinement. Mother’s conflicting feelings towards their child did not provide significant improved variance*.*

#### Mothers Psychological QoL

During step one of the regression analysis the enter method was used wherein the covariates: mothers’ education level and mothers’ psychological distress were added. In step two forward selection was used wherein mothers’ conflicting feelings towards her child was entered first (*r* =  − 0.36, *p* < 0.01), followed by feelings of confinement in their motherly role (*r* =  − 0.35, *p* < 0.01). Results show that 23% of the variance in mothers psychological QoL could be explained by mothers’ psychological distress [F(2,43) = 6.53, *p* =  < 0.01]. Another 8% of variance in mothers psychological QoL could be explained by mothers’ conflicting feelings towards their child [F(1,42) = 7.76, *p* = 0.04]. The best fitting models were found for mothers’ conflicting feelings towards their child. Mothers’ role confinement did not provide significant improved variance*.*

#### Mothers Social QoL

During step one of the regression analysis the enter method was used wherein the covariates: mothers education level and mothers’ psychological distress were added. In step two forward selection was used wherein mothers’ conflicting feelings towards her child was entered first (*r* =  − 0.36, *p* < 0.05), followed by feelings of confinement in their motherly role (*r* =  − 0.35, *p* < 0.01). Results show that 26% of the variance in mothers social QoL could be explained mothers’ psychological distress and their education level [F(2,43) = 7.50, *p* =  < 0.01]. Another 3% of variance in mothers psychological QoL could be explained by mothers’ conflicting feelings towards their child [F(1,35) = 4.31, *p* =  < 0.01]. The best fitting models were found for mothers’ psychological distress. Mothers’ conflicting feelings and role confinement did not provide significant improved variance*.* No interaction effect or curvilinearity was present.

#### Mothers Environmental QoL

During step one of the regression analysis the enter method was used wherein the covariates: mothers education level and mothers’ psychological distress were added. In step two forward selection was used wherein mothers’ feelings of confinement in their motherly role (*r* =  − 0.34, *p* < 0.01) was entered. Results show that 36% of the variance in mothers environmental QoL could be explained mothers’ psychological distress [F(2,43) = 11.86, *p* =  < 0.01]. The best fitting models were found for mother’s psychological distress. Mothers’ role confinement did not provide significant improved variance. No interaction effect or curvilinearity was present (Table 4).

**Table 4:** Mothers of a young child with ASD stepwise regression analysis to identify associations of QoL domains measured with the WHOQoL-BREF by maternal stress areas measured with the OBVL

	R	*R*^2^	Adjusted *R*^2^	Standard error of the estimate	Change statistics				
					*R*^2^ change	F change	df1	df2	Significant F change
Physical QoL									
Covariates	0.56	0.31	0.28	2.30	0.31	9.86	2	43	< 0.01
Covariates + role confinement	0.62^a^	0.38	0.34	2.20	0.07	5.03	1	42	0.03
Psychological QoL									
Covariates	0.48	0.23	0.20	1.91	0.23	6.53	2	43	< 0.01
Covariates + conflicting feelings	0.56^b^	0.31	0.26	1.83	0.08	4.76	1	42	0.04
Social QoL									
Covariates	0.51	0.26	0.22	2.25	0.26	7.50	2	43	< 0.01
Environmental QoL									
Covariates	0.60^a^	0.36	0.33	2.02	0.36	11.86	2	43	< 0.01

	Unstandardized coefficients		Standardized coefficients	t	Significance	95.0% Confidence interval for B	
	B	Standard error	β			Lower bound	Upper bound
Physical QoL							
(Constant)	27.39	2.74		9.99	0.00	21.86	32.92
Education	− 0.13	0.28	− 0.05	− 0.48	0.63	− 0.71	0.43
Psychological distress	− 0.18	0.04	− 0.48	− 3.92	0.00	− 0.27	− 0.08
Conflicting feelings	− 0.16	0.07	− 0.28	− 2.24	0.03	− 0.30	− 0.01
Psychological QoL							
(Constant)	23.70	2.27		10.40	0.00	19.10	28.30
Education	0.01	0.25	0.00	0.06	0.94	− 0.50	0.53
Psychological distress	− 0.12	0.03	− 0.41	− 3.16	0.01	− 0.20	− 0.04
Conflicting feelings	− 0.19	0.08	− 0.31	− 2.18	0.03	− 0.37	− 0.01
Social QoL							
(Constant)	24.18	2.77		8.70	0.01	18.58	29.79
Education	− 0.60	0.28	− 0.27	− 2.12	0.03	− 1.18	− 0.03
Psychological distress	− 0.14	0.04	− 0.41	− 3.13	0.01	− 0.24	− 0.05
Environmental QoL							
(Constant)	27.27	2.50		10.88	0.01	22.22	32.33
Education	− 0.30	0.258	− 0.14	− 1.17	0.24	− 0.82	0.21
Psychological distress	− 0.19	0.042	− 0.57	− 4.66	0.01	− 0.28	− 0.11

In step one, the covariates: education level and psychological distress were entered as mothers differed significantly from the norm population on these variables. In step two the maternal stress areas were added in ascending order of correlation with QoL

^a^Covariates + Role confinement and Conflicting feelings towards the child

^b^Covariates + Conflicting feelings towards the child and Role confinement

### Fathers QoL Associated with Paternal Stress Areas

#### Fathers Physical QoL

During step one of the regression analysis the enter method was used wherein the covariates: fathers’ marital status, education level, income and fathers’ psychological distress were added. In step two forward selection was used wherein stress derived from difficulties with parenting was entered first (*r* =  − 0.44, *p* < 0.01), followed by fathers’ conflicting feelings towards her child (*r* =  − 0.37, *p* < 0.01), and feelings of confinement in their fatherly role (*r* =  − 0.37, *p* < 0.01). Results show that 49% of the variance in fathers physical QoL could be explained fathers’ psychological distress [F(4,34) = 8.15, *p* =  < 0.01]. Another 14% of variance in fathers physical QoL could be explained by fathers’ conflicting feelings towards their child [F(1,33) = 12.01, *p* =  < 0.01]. The best fitting models were found for fathers’ conflicting feelings towards their child. Fathers’ difficulties with parenting and role confinement did not provide significant improved variance*.*

#### Fathers Psychological QoL

During step one of the regression analysis the enter method was used wherein the covariates: fathers’ marital status, education level, income and fathers’ psychological distress were added. In step two forward selection was used wherein stress derived from feeling confined in their fatherly role was entered first (*r* =  − 0.33, *p* < 0.05), followed by fathers’ conflicting feelings towards their child (*r* =  − 0.43, *p* < 0.01), and difficulties with parenting (*r* =  − 0.43, *p* < 0.01). Results show that 36% of the variance in fathers psychological QoL could be explained fathers’ psychological distress [F(4,34) = 4.68, *p* =  < 0.01]. Another 18% of variance in fathers psychological QoL could be explained by fathers’ difficulties with parenting their child [F(1,33) = 12.94, *p* =  < 0.01]. The best fitting models were found for fathers’ difficulties with parenting. Fathers’ conflicting feelings and feelings of role confinement did not provide significant improved variance*.*

#### Fathers Social QoL

During step one of the regression analysis the enter method was used wherein the covariates: fathers’ marital status, education level, income and fathers’ psychological distress were added. In step two forward selection was used wherein stress derived from conflicting feelings towards their child was entered (*r* =  − 0.38, *p* < 0.01). Results show that 55% of the variance in fathers social QoL could be explained fathers’ psychological distress and their education level [F(4,34) = 10.50, *p* =  < 0.01]. Another 10% of variance in fathers social QoL could be explained by fathers’ conflicting feelings towards their child [F(1,33) = 7.15, *p* = 0.01]. The best fitting models were found for fathers’ conflicting feelings.

#### Fathers Environmental QoL

During step one of the regression analysis the enter method was used wherein the covariates: fathers’ marital status, education level, income and fathers’ psychological distress were added. In step two forward selection was used wherein stress derived from fathers’ difficulties with parenting was entered first (*r* =  − 0.40, *p* < 0.01), followed by fathers’ conflicting feelings towards their child (*r* =  − 0.33, *p* < 0.05). Results show that 32% of the variance in fathers environmental QoL could be explained fathers own psychological distress [F(4,34) = 3.97, *p* = 0.01]. Another 8% of variance in fathers social QoL could be explained by fathers’ difficulties with parenting [F(1,33) = 4.17, *p* = 0.04]. The best fitting models were found for fathers’ difficulties with parenting (Table 5).

**Table 5:** Fathers of a young child with ASD stepwise regression analysis to identify associations of QoL domains measured with the WHOQoL-BREF by paternal stress areas measured with the OBVL

	R	R^2^	Adjusted R^2^	Standard error of the estimate	Change statistics				
					R^2^ change	F change	df1	df2	Significant F change
Physical QoL									
Covariates	0.70	0.49	0.43	1.66	0.49	8.15	4	34	< 0.01
Covariates + conflicting feelings	0.79^a^	0.63	0.57	1.44	0.14	12.01	1	33	< 0.01
Psychological QoL									
Covariates	0.60	0.36	0.28	1.96	0.36	4.68	4	34	< 0.01
Covariates + difficulties parenting	0.73^b^	0.54	0.47	1.69	0.18	12.94	1	33	< 0.01
Social QoL									
Covariates	0.74	0.55	0.50	2.31	0.55	10.51	4	34	< 0.01
Covariates + conflicting feelings	0.80^c^	0.63	0.58	2.13	0.08	7.15	1	33	< 0.05
Environment QoL									
Covariates	0.56	0.32	0.24	1.98	0.32	3.97	4	34	< 0.05
Covariates + difficulties parenting	0.63^d^	0.40	0.30	1.90	0.08	4.17	1	33	< 0.05

	Unstandardized coefficients		Standardized coefficients	t	Significance	95.0% Confidence interval for B	
	B	Standard error	β			Lower Bound	Upper Bound
Physical QoL							
(Constant)	18.01	1.24		14.55	0.00	15.54	20.59
Marital status	0.14	0.19	0.08	0.70	0.49	− 0.26	0.53
Education	0.23	0.37	0.07	0.61	0.54	− 0.53	0.98
I ncome	0.00	0.00	− 0.04	− 0.39	0.70	− 0.00	0.00
Psychological distress	− 2.08	0.42	− 0.58	− 4.99	0.00	− 2.92	− 1.23
Conflicting feelings	− 0.25	0.07	− 0.40	− 3.47	0.00	− 0.39	− 0.10
Psychological QoL							
(Constant)	19.84	1.78		11.15	0.00	16.22	23.47
Marital status	0.12	0.22	0.07	0.53	0.60	− 0.34	0.57
Education	− 0.05	0.44	− 0.01	− 0.18	0.92	− 0.95	0.86
Income	0.00	0.00	0.07	0.56	0.58	− 0.00	0.00
Psychological distress	− 1.66	0.48	− 0.44	− 3.47	0.00	− 2.64	− 0.69
Difficulties with parenting	− 0.26	0.07	− 0.46	− 3.60	0.00	− 0.40	− 0.11
Social QoL							
(Constant)	13.90	1.83		7.59	0.00	10.17	17.63
Marital status	0.33	0.29	0.14	1.13	0.27	− 0.26	0.91
Education	1.22	0.55	0.26	2.24	0.03	0.11	2.34
Income	0.00	0.00	0.08	0.72	0.48	− 0.00	0.00
Psychological distress	− 2.74	0.61	− 0.51	− 4.47	0.00	− 3.99	− 1.49
Conflicting feelings	− 0.28	0.10	− 0.30	− 2.67	0.01	− 0.49	− 0.07
Environment QoL							
(Constant)	17.59	2.00		8.79	0.00	13.52	21.66
Marital status	0.04	0.25	0.02	0.16	0.87	− 0.47	0.55
Education	0.54	0.50	0.16	1.08	0.29	− 0.48	1.56
Income	0.00	0.00	0.21	1.45	0.16	− 0.00	0.01
Psychological distress	− 1.20	0.54	− 0.32	− 2.23	0.03	− 2.30	− 0.10
Difficulties with parenting	− 0.16	0.08	− 0.30	− 2.04	0.05	− 0.33	− 0.00

In step one, the covariates: psychological distress, education, income, and marital status were entered as fathers differed significantly from the norm population on these variables. In step two the paternal stress areas were added in ascending order of correlation with QoL

^a^Covariates + Parenting difficulties, conflicting feelings towards the child

^b^Covariates + Role confinement, conflicting feelings towards the child and Difficulties with parenting

^c^Covariates + conflicting feelings towards the child

^d^Covariates + Difficulties with parenting and conflicting feelings towards the child

## Discussion

This study shows that high parental stress is already present in early parenthood, in which mothers and fathers display a different pattern. Their stress is closely related with specific QoL domains when raising a young child with ASD.

The current study indicates that already in the early years of raising children with ASD, parents experience higher levels of parental stress compared to mothers of typically developing children. These results are consistent with the limited research, which is done with respect to parenting children with ASD at this young age [18, 19, 23]. What is new, is that our study shows that high maternal stress derives from conflicting feelings towards their child, from feelings and thoughts concerning the challenges and difficulties encountered while parenting and from feeling confined in their general functioning due to their parenting role. While most studies focus on parental stress in general, our results provide specific insight into what parental stress stems from. Our results appear to be consistent with the increased parental stress resulting from unsatisfactory parent–child interactions and the child’s behavior characteristics which make him/her easy of difficult to manage [23], different parental stress questionnaires were used. As the PSI-sf [23] is an international questionnaire and the OBVL has been specifically developed for the Dutch population [33, 51].

In addition, this study explored differences between fathers and mothers showing that they display comparable parental stress from conflicting feelings towards their child and from difficulties with parenting their young child with ASD. Mothers experience more stress due to their parenting role than fathers. With respect to previous studies including both mothers and fathers, limited research explored both their stress levels, the studies that did, found contrasting results [3, 21, 24, 38, 42, 47]). For instance, some studies show that mothers and fathers were found to have similar levels of stress [21, 24] and others display that mothers had higher levels of stress than fathers [21, 24, 47]. Also, maternal stress from personal burdens such as poor health/mood, excessive time demands, or overprotection/dependency in comparison to fathers were identified [28, 38]. The contrasting results within these studies can be explained by the different age groups [21, 24, 38, 47], other instruments used PSI-4/SIPA [24], QRSFC [47] or QRS-S [38] or can be due to cultural differences (Western [24, 38] and Non-Western [47]). The difference in parental stress from feeling confined in their general functioning due to their parental role between mothers and fathers in this study can probably be explained by how mothers and fathers experience childcare. An important factor may be that Dutch mothers are often the primary caretaker of the child and will therefore be exposed to their child’s needs and problems on a more frequent basis than the other caretaker [6, 16] (https://www.nji.nl). Nevertheless, this study shows that both parents experience increased parental stress levels already at a young age of their child. As a result, fathers potentially have the same health risks as mothers. However, a recent study by van der Lubbe et al. [49] shows that although fathers and mothers of a young child with ASD both experience high parental stress levels, only mothers show more obesity, abdominal obesity, and metabolic syndrome than the general population [49]. An explanation for this can be that fathers and mothers use different forms of stress coping as is shown by Hastings et al. [25] where mothers reported more frequent use of active avoidance and problem-focused coping strategies than fathers [25]. While it is becoming increasingly clear that not only mothers, but also fathers of young children with ASD also experience high parental stress, although study designs did not always point to this comparison [3, 21, 24, 38, 42, 47]). In addition, the insight is emerging that fathers cope with their paternal stress differently than mothers [25] and this study points to the relation with their physical QoL. Research into their physical status including complaints of pain and health care use is needed to gain insight what risks these pose to fathers and how this can be addressed within the treatment of families with a child with ASD.

This study also explored the QoL in parents of young children with ASD. Our results show that parents experience lower QoL with respect to their psychological- and physical well-being, their social relationships and satisfaction with their living situation and other social economic factors compared to the general population. In addition, differences between mothers and fathers QoL were explored, showing that both mothers and fathers experience a lower QoL on all the QoL domains compared to the general population. These results are in line with an earlier study by Pisula & Porebowicz-Dorsmann (2017) comparing both mothers and fathers of older school aged children with ASD, showing that they both experience a lower satisfaction with their psychological- and physical well-being and social relationships than parents of typically developing children [38]. This study adds to our knowledge that the experience of low parental QoL already starts during the early years of parenting a child with ASD and that during this age also living situation and other social economic factors are important aspects of parental QoL. Looking at the relationship between parental stress and QoL, it appears that the extent to which mothers feel they are not being able to take care of themselves due to their role as a mother is related to how they perceive their physical health. While mothers' conflicting feelings towards their child associate with their psychological wellbeing. This is different for fathers; fathers' conflicting feelings towards their child relate to how satisfied they are with their physical health and social life, while fathers' difficulties with parenting are related to how satisfied they are with their living conditions. Thus, with respect to QoL, a -gender specific tendency seems to be present between maternal or paternal stress areas and their QoL domains. This could be an explanation why the effect of play-based interventions, which are often used in young children with ASD, on general parental stress is therefore variable [15]. As play-based interventions in children with ASD focus on parental stress in general, rather than on specific parental stress areas. This could argue in favour of looking at the stress levels of parents in different areas of functioning, for both mothers and fathers separately, before starting treatment.

### Strengths

Studies on parental stress in young children with ASD are underrepresented as most studies on parental stress and QoL include parents of children above the age of 7 years. Additionally, fathers are included in contrast to most studies on parental stress and QoL including only mothers.

### Limitations

The current study used a cross-sectional design, thus changes over time could not be observed. As the current study is part of on an ongoing RCT, we will provide these data in the future. Although the comparison of two populations with different cultural backgrounds (Dutch and Australian) is suboptimal and should be discussed in relation to interpretation of results, we conclude that given the similarities in government systems, cultural diversity, QoL ratings, and previous literature on the comparison of both populations, this comparison provides good insights into the QoL of the Dutch parents within our study population [37, 44]. Additionally, the mean age of children in this study is 4.6 years, however the age at which the diagnoses of ASD were received is unknown. This is a critical factor in interpreting parental stress, as research has shown that delays in diagnosis increase parental stress [35, 54]. The average age at which ASD is diagnosed in Dutch children < 18 is at 5.1 years old, almost equal to the average worldwide age of 5 years [4, 50]. Therefor the majority of the children and their parents in this study in all probability did not experience delays in diagnosis.

In conclusion, already at young age, parenting of children with ASD is a major challenge for both mothers and fathers, resulting in high stress levels and compromised QoL. Based on our findings we recommend that attention is given to physical, psychological, and social well-being of parents in the treatment of children with ASD. By doing so, both parents and child get the attention to optimally support the child's development.

## Summary

Parents of children with ASD are at risk for chronic stress due to challenging parenting. It is unknown whether stress is already present in early parenthood, similar for mothers and fathers and if this impacts quality of life (QoL). The present study explored whether high parental stress and low QoL was already present in both mothers (n = 56) and fathers (n = 51) of young children (aged 3 to 7) with ASD and if maternal and paternal stress impacted parental QoL. Associations between parental stress and QoL were examined by questionnaires: the parenting burden questionnaire (in Dutch: *Opvoedbelasting Vragenlijst*) and the World Health Organization Quality of Life (abridged version) questionnaire. This study showed that high parental stress from conflicting feelings towards their child and from difficulties with parenting was already present in early parenthood. Mothers and fathers displayed a different pattern, where mothers experience more stress due to their parenting role than fathers. This observation might be explained by how mothers and fathers experience childcare and that Dutch mothers are often the primary caretaker of their child with ASD, and will therefore be exposed to their child’s needs and problems on a more frequent basis than fathers. Also, the experience of low QoL already started during the early years of parenting a child with ASD as both fathers and mothers of young children with ASD reported lower QoL with respect to their psychological- and physical well-being, their social relationships and satisfaction with their living situation and other social economic factors compared to the general population. Their parentings stress was closely related with specific QoL domains in which also a gender specific tendency was present between maternal or paternal stress areas and their QoL domains. Increased maternal conflicting feelings towards the child associated with lower psychological QoL, while high maternal feelings of role confinement associated with low physical QoL. Increased paternal conflicting feelings towards their child related to lower physical and social QoL, while fathers with more parenting difficulties reported less satisfaction with their psychological and environmental wellbeing. Thus, already at young age, parenting children with ASD is a major challenge for both mothers and fathers. Our study argues in favour of examining stress levels of parents in different areas of functioning, for both mothers and fathers separately, before starting treatment. Additionally, attention has to be given to physical, psychological, and social well-being of parents in the treatment of children with ASD. By doing so, both parents and child get the attention to optimally support the their child's development.

## Data Availability

Not applicable.

## Appendix 1: Interpretation Framework for the Parental Stress Questionnaire (OBVL)

**Table:**

T-scores	Percentile	Label	Meaning	Implication
< 60	< 84	Normal range	No problems	No considerable worries
60–63	85–90	Subclinical range	Mild problems	Problems deserve attention
64–66	91–95	Clinical range	Considerable problems	Problems deserve treatment
67–69	96–97		Severe problems	
> 70	> 98		Very severe problems	

This interpretation framework is derived from the parental stress questionnaire (OBVL) manual [51]

## Appendix 2: Interpretation Framework for Parental Quality of Life Measured with the WHOQoL-BREF

**Table:**

	n	Health status	Physical		Psychological		Social		Environment	
			Mean	(sd.)	Mean	(sd.)	Mean	(sd.)	Mean	(sd.)
Women	481	Excellent	89.0	(9.2)	82.0	(9.3)	82.0	(13.5)	83.9	(11.1)
		Very good	82.1	(11.7)	74.5	(11.4)	75.8	(16.6)	77.6	(12.0)
		Good	72.1	(14.2)	69.1	(10.5)	70.9	(17.1)	74.6	(12.5)
		Fair	61.8	(14.7)	64.4	(13.7)	71.3	(17.4)	70.5	(12.7)
		Poor	45.7	(18.6)	56.2	(13.6)	61.3	(21.4)	63.1	(12.9)
Men	376	Excellent	93.7	(7.7)	86.9	(11.8)	81.3	(15.5)	83.8	(11.1)
		Very good	83.3	(12.3)	76.0	(11.2)	72.9	(17.0)	79.5	(10.4)
		Good	75.8	(12.7)	70.3	(11.9)	69.8	(16.4)	75.4	(11.7)
		Fair	60.7	(15.3)	63.2	(12.1)	61.5	(20.9)	67.8	(12.6)
		Poor	46.2	(19.9)	55.5	(17.9)	57.4	(21.1)	67.4	(13.0)

This interpretation framework is derived from an Australian norm population [26]
